# Early peritoneal lavage with ulinastatin improves outcome and enhances multi-organ protection in a model of severe acute pancreatitis

**DOI:** 10.3892/etm.2015.2251

**Published:** 2015-02-03

**Authors:** CONG FENG, XUAN SU, XUAN ZHOU, LI-LI WANG, BEI LI, LI CHEN, FA-QIN LV, TAN-SHI LI

**Affiliations:** 1 Department of Emergency, General Hospital of the PLA, Beijing 100853, P.R. China; 2 The Li-Shi Road Outpatient Department of the General Hospital of the Second Artillery, Beijing 100045, P.R. China; 3 Department of Hepatology-Immunology, Beijing You An Hospital of the Capital Medical University, Beijing 100069, P.R. China; 4 Department of Ultrasound, General Hospital of the PLA, Beijing 100853, P.R. China

**Keywords:** early stage, peritoneal lavage, antiprotease, severe acute pancreatitis, ulinastatin

## Abstract

The aim of this study was to investigate the effect of early peritoneal lavage with ulinastatin on the outcome of a rat model of severe acute pancreatitis (SAP). A total of 80 male Wistar rats were randomly divided into the following groups: Sham-operated (C), SAP model (M), saline lavage (SL), intravenous ulinastatin (IU), early ulinastatin lavage (EUL) and late ulinastatin lavage (LUL). Intraperitoneal lavage or injection were performed immediately subsequent to the establishment of the SAP model in groups SL, IU and EUL and 3 h later in group LUL. Intraperitoneal lavage with or without ulinastatin was performed for 3 h. The survival time of the rats in groups C, M, EUL and LUL was recorded over a 12-h period and the median survival time was calculated. At 3 h after the induction of SAP, histopathological analyses were performed and the biochemical parameters of groups C, M, SL, IU and EUL were assessed. Groups EUL and LUL exhibited an increased median survival time compared with Group M, with the survival time of the rats in group EUL markedly longer than that in the group LUL rats. Group SL, IU and EUL rats were found to have reduced plasma activities of amylase, lipase, aspartate transaminase and alanine transaminase, with the biggest change observed in the group EUL rats. Furthermore, the intervention in groups SL and EUL was more effective at reducing creatinine and urea levels than that in group IU. Rats in group EUL exhibited a greater inhibition of the SAP-induced increase in troponin T levels than rats in groups SL and IU. The pathological severity scores of the pancreas, liver, kidney and lung in group EUL were significantly lower than those in groups M and better than those in groups SL and IU. In conclusion, early intraperitoneal lavage with ulinastatin significantly improves the median survival time and protects multi-organ function in an SAP model.

## Introduction

Peritoneal antiprotease lavage therapy for pancreatitis has been investigated for a number of years, but the results of experimental and clinical studies have been conflicted. Intraperitoneal lavage therapy with added protease inhibitors, including camostate and glutaryl-trialanin-ethylamide, has been proven to improve survival rate in several species with experimentally induced severe acute pancreatitis (SAP) (1–3). However, certain studies in humans have not shown a significant improvement in survival rate with peritoneal antiprotease lavage therapy (4–6). Between 40 and 60% of in-hospital patient mortalities occurring within one week of admission are associated with early multi-organ failure in SAP (7). Early-stage intervention, including peritoneal lavage for SAP, could significantly improve this outcome (8,9), and the early treatment of SAP, would play a key role in avoiding high mortalities rates. Peritoneal antiprotease lavage therapy in the early stages of SAP may achieve a superior therapeutic effect to late-stage lavage therapy.

Ulinastatin is a purified antiprotease obtained from the fresh urine of healthy adults has been shown to exert significant therapeutic effects in several forms of acute pancreatitis (10,11); however, the effect of peritoneal ulinastatin lavage in the early stages of SAP has not been studied. According to our preliminary experiment, peritoneal lavage with 62.5 U/ml ulinastatin added to the lavage fluid exerted the best therapeutic effect. The aim of the present study was therefore to use a rat model to investigate the effect of intraperitoneal ulinastatin lavage in the early stages of SAP on the multi-organ protection and overall outcome, as well as to provide experimental and theoretical evidence for the treatment of SAP in the clinical setting.

## Materials and methods

### Experimental animals

A total of 80 healthy male Wistar rats (weight, 300±15 g) were obtained from the Experimental Animal Center of the General Hospital of the PLA (Beijing, China). The experimental protocol was approved by the Ethics Committee for Animal Research from the General Hospital of the PLA and all experimental rats received humane care.

### Reagents

The reagents used in the study were purchased from the following companies: Chloral hydrate (Shanghai Yingxin Laboratory Equipment Co., Ltd., Shanghai, China); sodium taurocholate (Shanghai Hufeng Biotechnology Co., Ltd., Shanghai, China); and ulinastatin (Guangdong Tianpu Biochemical Pharmaceutical Co., Ltd., Guangzhou, China). The amylase and lipase assay kit was purchased from Shanghai Shifeng Biotechnology Co., Ltd. (Shanghai, China); the aspartate transaminase (AST) and alanine transaminase (ALT) kit was obtained from Shanghai Hu Ding Biotechnology Co., Ltd. (Shanghai, China); the creatinine (Cr) and urea (UR) assay kit was purchased from Beijing Bomaisi Biological Technology Co., Ltd. (Beijing, China) and the troponin T (TnT) assay kit was purchased from Shanghai Ji Ning Industrial Co., Ltd. (Shanghai, China).

### Experimental groups

The rats were randomly divided into six groups: Group C (n=18, control group), sham surgery without the induction of SAP or peritoneal lavage/intravenous injection, but with catheter insertion; group M (n=18, SAP model group), induction of SAP without peritoneal lavage or intravenous injection, but with catheter insertion; group SL (n=8, saline lavage group), induction of SAP with saline lavage; group IU (n=8, intravenous ulinastatin group), intravenous ulinastatin (2,500 U/100 g) immediately subsequent to the induction of SAP, with catheter insertion but without peritoneal lavage; group EUL (n=18, early ulinastatin lavage group), ulinastatin (62.5 U/ml) lavage immediately subsequent to the induction of SAP; group LUL (n=10, late ulinastatin lavage group), ulinastatin (62.5 U/ml) lavage 3 h after the induction of SAP.

### Animal model

The rats were fasted for 12 h and had no water for 4 h prior to surgery. The rats were anesthetized with intraperitoneal injections of 10% chloral hydrate (3 ml/kg). Subsequent to making an incision in the abdomen and clamping the distal region of the duodenal bile duct with injury-free metal clips, a syringe needle was inserted into the opening of the duodenal bile duct and 5% sodium taurocholate (freshly prepared in saline solution, 0.6 ml) was retrogradely injected into the duct at constant rate of 0.2 ml/min using an infusion pump. After 5 min, the needle and metal clips were removed. A consistently high mortality rate (>80% within 12 h) was obtained. Group C rats underwent sham surgery without the induction of SAP.

Prior to the closure of the abdomen, a silicon catheter (catheter A) with five lateral outlets was placed adjacent to the pancreas and another silicon catheter (catheter B) with five lateral outlets was placed in the pelvic cavity. All groups underwent peritoneal catheter insertion.

### Peritoneal lavage

Intraperitoneal lavage was performed immediately subsequent to the establishment of the SAP model in groups SL and EUL and 3 h later in group LUL. Warmed (37°C) lavage fluid was injected from catheter A at 80 ml/h for 15 min and catheter B was blocked. Following this, catheter A was blocked and fluid was allowed to flow out for 15 min from catheter B. Each lavage procedure thus lasted 30 min, and the lavage was performed six times (for 3 h in total) (1). The volume input and output were monitored. The lavage fluid consisted of saline solution with or without the addition of 62.5 U/ml ulinastatin. This concentration of ulinastatin was shown to exert the best therapeutic effect in our preliminary studies. Following lavage, catheters A and B were blocked and the rats were kept in single cages with free access to water but no solid food.

### Intravenous ulinastatin

To compare the effect of peritoneal lavage with that of intravenous ulinastatin administration, group IU rats were administered intravenous ulinastatin at 2,500 U/100 g (freshly prepared in 0.15 ml saline solution, approximately equivalent to the total dose applied in groups EUL and LUL) to the caudal vein immediately subsequent to SAP induction. These rats did not undergo lavage.

### Assays and calculations

The survival times of the rats in groups C, M, EUL and LUL (n=10 per group) were recorded over a 12-h period and the median survival time was calculated. Animals surviving to 12 h were anesthetized and sacrificed. Rats in groups C, M, SL, IU and EUL (n=8 per group) were sacrificed for histopathological analyses and biochemical parameter (amylase, lipase, AST, ALT, CR, UR and TnT) measurements 3 h after the establishment of each model. As the rats in group LUL did not receive the intervention (ulinastatin lavage) until 3 h following the induction of the SAP model, histological analysis was not performed as the results would be incomparable to the other groups due to the difference in time. Arterial blood was additionally collected into heparinized syringes from the abdominal aorta following general anesthesia and a second laparotomy. The biochemical parameter measurements were conducted using an automatic biochemical analyzer (Beckman Coulter-AU5800; Beckman Coulter, Brea, CA, USA).

The organs selected for histological examination (pancreas, liver, kidney and lung) were fixed in formalin, subjected to conventional dehydration and embedded in paraffin. The samples were then cut into 5-μm sections and stained with hematoxylin and eosin. Examination by light microscopy was performed by two professional pathologists using a double-blind method as to whether the section was from the control or one of the treatment groups. Three slices were randomly selected for each group; for each slice, 10 high-power fields of vision were again randomly selected. The pathological score was calculated according to the methods described by Zhang *et al* (12–14).

### Statistical analysis

Data are expressed as the mean (standard deviation) for normally distributed variables or as the median (interquartile range) for highly skewed variables. Statistical analyses were performed using the SPSS 19.0 software package (IBM-SPSS, Armonk, NY, USA).

In the survival experiments, analysis of the median survival time at the end of the 12-h observation period was conducted by the Kaplan-Meier or Kruskal-Wallis H tests. Analysis of variance was used for the comparison of normally distributed data. Multiple comparisons were subjected to Kruskal-Wallis H and Bonferroni correction tests. The χ^2^ test was used to evaluate the equality of frequencies for discrete variables. P<0.05 was considered to indicate a statistically significant difference.

## Results

### Effect of early peritoneal lavage with ulinastatin on the survival time

**Figure 1:**
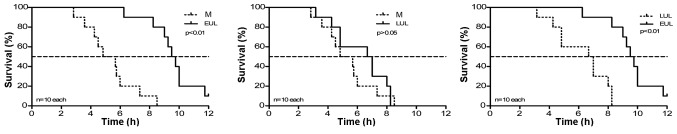
Effect of early and late peritoneal lavage with ulinastatin on the survival time of rats. The survival times of rats in groups C, M, EUL and LUL (n=10 per group) was recorded over a 12-h period and median survival time was calculated. Early peritoneal ulinastatin lavage significantly improved the median survival time compared with group M (P<0.01), but late lavage did not (P>0.05). Early peritoneal ulinastatin lavage exerted a superior effect to late lavage (P<0.01). Early peritoneal ulinastatin lavage was performed immediately subsequent to the establishment of the SAP model and the late peritoneal ulinastatin lavage was performed 3 h later. All peritoneal lavage with ulinastatin was performed for 3 h. Group M, SAP model group; group EUL, early ulinastatin lavage group; group LUL, late ulinastatin lavage group; SAP, severe acute pancreatitis.

All rats in group C were alive at 12 h. The median survival time of the group M rats was 4.83 h. The survival time of rats in group EUL (ulinastatin lavage was performed immediately subsequent to the induction of SAP) was significantly longer than that of the group M rats (9.50 vs. 4.83 h). Group LUL rats (ulinastatin lavage was performed at 3 h after induction of SAP) also exhibited an increased median survival time compared with the group M rats (6.67 vs. 4.83 h), but the difference was not significant (P>0.05). Early ulinastatin lavage therefore improved the prognosis of the SAP rats to a greater extent than the late lavage (9.50 vs. 6.67 h). The results are summarized in Table I and Fig. 1.

### Effect of early peritoneal lavage with ulinastatin on multi-organ functional protection

**Figure 2:**
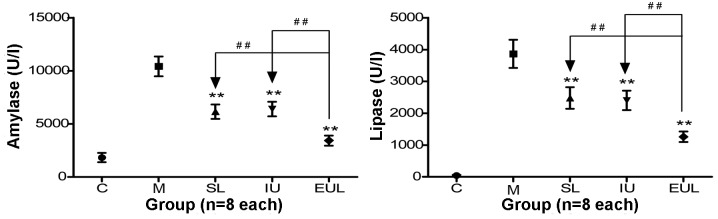
Effect of early peritoneal ulinastatin lavage on the activity of pancreatic enzymes (amylase and lipase). Rats in groups C, M, SL, IU and EUL (n=8 per group) were sacrificed for amylase and lipase measurements 3 h after the establishment of each model. The plasma amylase and lipase activity in group M was significantly higher than that in group C. The amylase and lipase activity in groups SL, IU and EUL was significantly reduced compared with that in group M, with the biggest reduction observed in group EUL. Results are presented as the mean ± standard deviation. ^**^P<0.01 vs. group M; ^##^P<0.01. Group C, control (sham-operated) group; group M, severe acute pancreatitis model group; group SL, saline lavage group; group IU, intravenous ulinastatin group; group EUL, early ulinastatin lavage group.

The analysis of the pancreatic enzyme activity showed that the activity of amylase and lipase in the plasma of rats in group M was significantly higher than that in the plasma of rats in group C. Furthermore, the amylase activity in groups SL, IU and EUL was significantly reduced compared with that in group M, with the greatest reduction observed in group EUL. The lipase activity in groups SL, IU and EUL was also significantly reduced compared with that in group M, and the reduction was similarly most marked in group EUL (Table II and Fig. 2).

**Figure 3:**
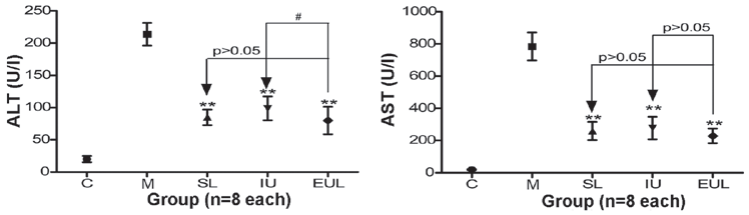
Effect of early peritoneal ulinastatin lavage on the activity of liver enzymes (ALT and AST). Rats in groups C, M, SL, IU and EUL (n=8 per group) were sacrificed for ALT and AST measurements 3 h after the establishment of each model. The plasma ALT and AST activity in group M was significantly increased compared with that in group C. The ALT and AST activity in groups SL, IU and EUL was significantly reduced compared with that in group M. The activity of ALT in group EUL was significantly lower than that in group IU, but the AST activity in group EUL was not significantly different from that in groups IU or SL. Results are presented as the mean ± standard deviation. ^**^P<0.01 vs. group M; ^#^P<0.05. Group C, control (sham-operated) group; group M, severe acute pancreatitis model group; group SL, saline lavage group; group IU, intravenous ulinastatin group; group EUL, early ulinastatin lavage group; ALT, alanine transaminase; AST, aspartate transaminase.

The analysis of liver enzyme activity showed that the plasma ALT and AST activity in Group M was significantly increased compared with that in group C. Compared with group M, groups SL, IU and EUL exhibited significantly reduced ALT activity. Furthermore, the ALT activity in group EUL was significantly lower than that in group IU. The AST activity in groups SL, IU and EUL was also significantly reduced compared with that in group M, but no significant difference was found among the results for groups SL, IU and EUL (Table II and Fig. 3).

**Figure 4:**
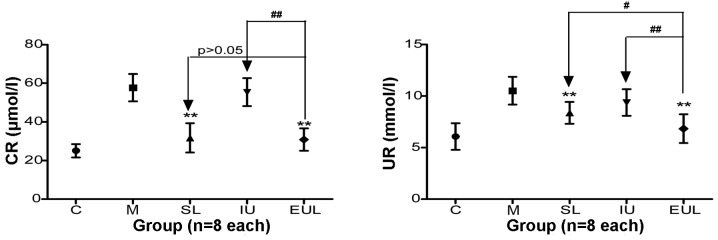
Effect of early peritoneal ulinastatin lavage on the CR and UR levels in the kidney. Rats in groups C, M, SL, IU and EUL (n=8 per group) were sacrificed for CR and UR measurements 3 h after the establishment of each model. The plasma CR and UR levels in group M were significantly increased compared with those in group C. The CR and UR levels in groups SL and EUL were significantly lower than those in group M and better than those in group IU. The effect in group EUL was superior to that in other groups. Results are presented as the mean ± standard deviation. ^**^P<0.01 vs. group M; ^#^P<0.05 and ^##^P<0.01. Group C, control (sham-operated) group; group M, severe acute pancreatitis model group; group SL, saline lavage group; group IU, intravenous ulinastatin group; group EUL, early ulinastatin lavage group; CR, creatinine; UR, urea.

The plasma CR and UR levels in the kidney in group M were significantly increased compared with those in group C. Compared with the CR level in group M, the CR level in groups SL and EUL was reduced to a greater extent than that in group IU; no significant difference was found between the results for groups SL and EUL. The UR level in groups SL and EUL was significantly reduced compared with that in group M and was lower than that in group IU (Table II and Fig. 4).

**Figure 5:**
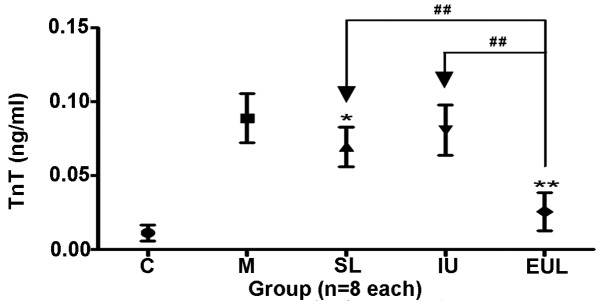
Effect of early peritoneal ulinastatin lavage on the TnT level in the heart. Rats in groups C, M, SL, IU and EUL (n=8 per group) were sacrificed for the measurement of the TnT level 3 h after the establishment of each model. The plasma TnT level in group M was significantly higher than that in group C. The plasma TnT level in groups EUL and SL was significantly lower that in groups M and IU. No significant difference was found between groups IU and M. Results are presented as the mean ± standard deviation. ^*^P<0.05 and ^**^P<0.01 vs. group M; ^##^P<0.01. Group C, control (sham-operated) group; group M, severe acute pancreatitis model group; group SL, saline lavage group; group IU, intravenous ulinastatin group; group EUL, early ulinastatin lavage group; TnT, troponin T.

The plasma TnT level in the heart in group M was significantly increased compared with that in group C. The intervention in groups EUL and SL effectively inhibited the increase in TnT observed in group M, but the intervention in group IU did not. (Table II and Fig. 5).

### Effect of early peritoneal lavage with ulinastatin on the pathological severity scores of multiple organs

The pathological severity scores of the pancreas, liver, kidney and lung in group M were significantly higher than those in group C; however, the scores in group EUL were significantly lower than those in group M. The pathological severity scores of the pancreas, liver, kidney and lung in the group EUL showed a greater improvement than those in groups SL and IU. The results are summarized in Table III.

## Discussion

Peritoneal antiprotease lavage therapy has been the focus of experimental and clinical research in pancreatitis for decades. It has been well established that the release of pancreatic enzymes into the peritoneal exudate is extremely toxic and can lead to multi-organ damage and death; at such times, peritoneal antiprotease lavage becomes necessary and should be directed into the peritoneal cavity for the best therapeutic effect (4). Based on this theory, numerous experimental and clinical studies concerning peritoneal antiprotease lavage therapy have been performed. Experimental studies have predominantly shown that peritoneal antiprotease lavage improves the SAP-related mortality rate and outcome (1,15), whereas clinical studies have yielded more conflicting results for unknown reasons (16,17).

It has been found that 40–60% of in-hospital patient mortalities occurring within one week of admission are associated with early multi-organ failure in SAP (7). Early intervention, including peritoneal lavage for SAP, could significantly improve the early prognosis (8,9). The stage at which peritoneal antiprotease lavage therapy is performed during the course of SAP may therefore be an important factor in achieving the optimal therapeutic effect.

Ulinastatin is a purified glycoprotein obtained from the fresh urine of healthy adult males. Ulinastatin has been suggested to have a considerable therapeutic effect in SAP, as it can suppress the pathogenesis and development of pancreatitis by inhibiting pancreatic enzyme activation. Furthermore, the therapeutic effects of ulinastatin are believed to be superior to those of aprotinin (10). The administration of ulinastatin through an arterial infusion catheter route has been suggested to result in enhanced therapeutic efficacy compared with intravenous administration, since only a small proportion of the administered ulinastatin reaches the pancreas with intravenous application (18,19). To date, there have been no experimental studies reporting the effect of early peritoneal ulinastatin lavage for SAP, which is a significant obstacle for a commitment to a clinical study.

On the basis of our preliminary experiment, peritoneal lavage with 62.5 U/ml ulinastatin added to the lavage fluid was shown to be most beneficial on the outcome of SAP. To the best of our knowledge, the present study is the first to evaluate the effect of early versus late peritoneal ulinastatin lavage on the median survival time in an SAP model. The effect of the early peritoneal ulinastatin lavage on multi-organ protection was also compared with the effect of intravenous ulinastatin administration.

In the present study, a retrograde injection of 5% sodium taurocholate (freshly prepared in saline solution, 0.6 ml) was applied into the pancreatic duct at a rate of 0.2 ml/min to produce an SAP experimental model with a high mortality rate (>80% within 12 h) (12). This high-mortality SAP model was selected for the study as it reflects life-threatening pancreatitis in humans. The results showed that early peritoneal ulinastatin lavage significantly improved the median survival time of the rats and was superior to late lavage. This indicates that peritoneal ulinastatin lavage could show efficacy at improving outcomes when performed in the early stages of SAP, but not in the late stages of the condition. A relevant clinical study showed that early fluid resuscitation was associated with a reduced incidence of systemic inflammatory response syndrome and organ failure (20). Peritoneal lavage may therefore replicate the role of fluid resuscitation, which is important for the prognosis in the early stage of the condition, and may enable ulinastatin to exert its optimal therapeutic effect directly and act locally on the pancreas and its associated enzymes in the peritoneal cavity. This theory was supported by the present results, which showed that the activity of amylase and lipase was reduced to a greater extent in group EUL than that in the group with intravenous administration; however, further studies are required to verify this theory.

The present study showed that, in the early stages of SAP, saline lavage, intravenous ulinastatin administration and peritoneal ulinastatin lavage could all reduce the activity of the liver enzymes AST and ALT. The results indicated that peritoneal lavage with or without ulinastatin may have been more effective than the intravenous route, but no significant difference was observed. Intravenous ulinastatin administration achieved higher therapeutic efficacy in the liver than in the pancreas; this may have been because intravenous administration led to the accumulation of the compound in the liver, but did not deliver sufficient quantities to the pancreas (18). The results suggested that ulinastatin could protect liver function and exert a superior therapeutic effect when administered via a peritoneal lavage route.

Plasma UR and CR levels in the kidney were reduced more effectively with peritoneal lavage than with intravenous administration, irrespective of whether the lavage fluid contained ulinastatin. Although ulinastatin can accumulate in the kidney through intravenous injection, the fluid resuscitation provided by the peritoneal lavage may play a more vital role in organ protection in the early stages of SAP. It is therefore possible that the significant improvement in the circulation induced by intraperitoneal lavage may be responsible for the reduction in the levels of UR and CR.

The results of the present study additionally showed that ulinastatin administration via peritoneal lavage was more effective at reducing the plasma TnT level in the heart than peritoneal lavage without ulinastatin or intravenous ulinastatin administration. Comparison of the TnT level in groups EUL (peritoneal ulinastatin lavage) and SL (peritoneal saline lavage) indicated that ulinastatin could have a protective effect on the heart, while comparison of groups EUL and IU showed that the protective effect of ulinastatin was enhanced with peritoneal administration. Peritoneal ulinastatin lavage in the early stages of SAP is therefore likely to have a significant beneficial effect on the prognosis.

The survival rate of the rats in group EUL at 12 h (one out of 10) was not significantly improved compared that in group M (zero out of 10), which may have been due to the short-term nature of the peritoneal lavage and insufficient fluid resuscitation. It has been reported that early and extended peritoneal lavage may be a useful therapy in the management of SAP (21). Early and target-oriented fluid resuscitation remains crucial in the early stages of SAP, although the peritoneal lavage may exert a similar effect (22,23). Peritoneal ulinastatin lavage in the early stages of SAP is therefore a promising strategy, as it not only replicates fluid resuscitation but also enhances the antiprotease effect. The early administration of ulinastatin by peritoneal lavage may be worthy of a clinical trial to ascertain whether pancreatitis-associated multiple organ dysfunction can be prevented or reduced in order to improve the outcome of patients with SAP.

## Figures and Tables

**Table I:** Comparison of the effect of early and late peritoneal ulinastatin lavage on survival time.

				P-value	
				
Group	n	Median survival time (h)	Interquartile range	Compared with group M	Compared with group LUL
C	10	12.00			
M	10	4.83	2.83–8.50		
LUL	10	6.67	3.17–8.25	0.45	
EUL	10	9.50	6.25–12.00	<0.01	<0.01

The survival time of rats in groups C, M, EUL and LUL (n=10 per group) was recorded for 12 h and the median survival time was calculated. Group M, severe acute pancreatitis model group; group EUL, early ulinastatin lavage group; group LUL, late ulinastatin lavage group.

**Table II:** Effect of different treatments on multi-organ protection.

Parameter	C	M	SL	IU	EUL
Pancreas					
Amylase, U/l	1831.1 (437.60)^a^	10422.11 (937.18)	6151.84 (681.31)^a^	6400.05 (678.65)^a^	3426.76 (484.43)^a^
Lipase, U/l	42.8 (21.40)^a^	3864.43 (443.13)	2476.95 (336.20)^a^	2403.51 (304.51)^a^	1261.34 (166.34)^a^
Liver					
ALT, U/l	20.00 (4.83)^a^	214.04 (17.58)	84.85 (12.15)^a^	98.61 (18.50)^a^	79.86 (21.39)^a^
AST, U/l	20.33 (3.35)^a^	783.99 (86.99)	259.26 (56.53)^a^	277.33 (69.77)^a^	229.15 (45.78)^a^
Kidney					
CR, μmol/l	25.09 (3.42)^a^	57.73 (7.06)	31.70 (7.60)^a^	55.39 (7.23)	30.85 (5.82)^a^
UR, mmol/l	6.07 (1.30)^a^	10.52 (1.34)	8.37 (1.06)^a^	9.37 (1.30)	6.83 (1.39)^a^
Heart					
TnT, ng/ml	0.011 (0.005)^a^	0.089 (0.017)	0.070 (0.014)^b^	0.081 (0.017)	0.026 (0.013)^a^

At 3 h after the establishment of each model, rats in groups C, M, SL, IU and EUL (n=8 per group) were anesthetized and sacrificed. Values are presented as the mean (standard deviation).

a P<0.01 and

b P<0.05, compared with group M.

Group C, control (sham-operated) group; group M, severe acute pancreatitis model group; group SL, saline lavage group; group IU, intravenous ulinastatin group; group EUL, early ulinastatin lavage group; ALT, alanine transaminase; AST, aspartate transaminase; CR, creatinine; UR, urea; TnT, troponin T.

**Table III:** Comparison of the pathological severity scores in multiple organs.

Organ	C	M	SL	IU	EUL
Pancreas	0.0 (0.0)^a^	12.0 (2.0)	9.0 (2.0)^a^	8.0 (2.0)^a^	7.0 (2.0)^a^
Liver	0.0 (0.0)^a^	1.0 (1.0)	0.5 (1.0)	0.5 (1.0)	0.0 (1.0)
Kidney	0.0 (0.0)^a^	2.0 (1.0)	1.0 (1.0)	2.0 (1.0)	1.0 (0.3)^b^
Lung	0.0 (0.0)^a^	2.0 (1.0)	2.0 (1.0)	1.0 (0.5)^b^	1.0 (1.0)^b^

Rats in groups C, M, SL, IU and EUL (n=8 were group) were sacrificed for histopathological analyses. The pathological score was calculated according to the method described by Zhang *et al* (7–9). Values are presented as the median (standard deviation).

a P<0.01 and

b P<0.05, compared with group M.

Group C, control (sham-operated) group; group M, severe acute pancreatitis model group; group SL, saline lavage group; group IU, intravenous ulinastatin group; group EUL, early ulinastatin lavage group.
